# Correlation between Lymphocyte-to-Monocyte Ratio (LMR), Neutrophil-to-Lymphocyte Ratio (NLR), Platelet-to-Lymphocyte Ratio (PLR) and Tumor-Infiltrating Lymphocytes (TILs) in Left-Sided Colorectal Cancer Patients

**DOI:** 10.3390/biology11030385

**Published:** 2022-02-28

**Authors:** Cieszymierz Gawiński, Wojciech Michalski, Andrzej Mróz, Lucjan Wyrwicz

**Affiliations:** M. Skłodowska-Curie National Research Institute of Oncology, ul. Wawelska 15, 02-034 Warsaw, Poland; wojciech.michalski@pib-nio.pl (W.M.); andrewcio@wp.pl (A.M.); lucjanwyrwicz@gmail.com (L.W.)

**Keywords:** lymphocyte-to-monocyte ratio (LMR), neutrophil-to-lymphocyte ratio (NLR), platelet-to-lymphocyte ratio (PLR), tumor-infiltrating lymphocyte (TIL), colorectal cancer (CRC)

## Abstract

**Simple Summary:**

Colorectal cancer (CRC) is one of the most common cancers worldwide. Novel markers have been investigated in order to better predict the course of disease and adjust the treatment. Markers associated with cancer-related inflammation (CRI), both in the bloodstream and the tumor tissue, have been in the spotlight for years. In this study, we investigate whether blood-based markers: lymphocyte-to-monocyte ratio, neutrophil-to-lymphocyte ratio or platelet-to-lymphocyte ratio, correlate with tissue-based markers, such as tumor-infiltrating lymphocytes. We retrospectively analyzed 87 patients with locally advanced left-sided CRC treated with radical surgery. Fifty patients were found suitable for the study. We compared the results of their blood tests from the time of the surgical intervention and the density of lymphocytes in the resected tumors. We found no correlation between local and peripheral markers of CRI. Further prospective studies are needed to confirm the results.

**Abstract:**

Colorectal cancer (CRC) is one of the leading causes of cancer-related mortality worldwide. Novel markers are required in order to select high-risk patients and better adjust the treatment. Both peripheral and local markers of cancer-related inflammation (CRI) such as lymphocyte-to-monocyte ratio (LMR), neutrophil-to-lymphocyte ratio (NLR) or platelet-to-lymphocyte ratio (PLR) and tumor-infiltrating lymphocytes (TILs) have been thoroughly investigated in recent years and deemed to be highly prognostic. We hypothesized that there is an association between local and peripheral CRI indices and that blood-based biomarkers may serve as a surrogate of TILs. We retrospectively analyzed 87 patients with locally advanced left-sided CRC treated with radical-intent surgery in the Maria Skłodowska-Curie National Research Institute of Oncology in Warsaw, Poland, between January 2014 and December 2015. Fifty patients were found eligible for the study. The patients were divided in terms of pre-treatment values of systemic inflammatory response (SIR) markers into LMR/NLR/PLR-high and low groups. We evaluated the resected specimens by immunohistochemistry in order to assess the densities of CD3+ and CD8+ lymphocytes in the center of the tumor and in the invasive margin. We found that the level of CD3+ lymphocytes in the center of the tumor was statistically significantly higher in patients with low pre-treatment NLR (*p* = 0.044); however, no correlation between any of the SIR markers and CD3+ or CD8+ TILs was observed. Five-year overall survival (OS) was longer in patients with high LMR (*p* < 0.001), low NLR (*p* = 0.001) and low PLR (*p* = 0.095). No correlation between the density of TILs and OS was demonstrated. In conclusion, based on our study, peripheral blood-based markers and CD3+ and CD8+ TILs are not interrelated.

## 1. Introduction

Colorectal cancer is the third most common cancer and the second most common cause of cancer-related death worldwide [1]. Despite advances in surgical procedures and adjuvant chemotherapy, approximately 20% of patients still experience relapse following curative treatment [2]. The Union for International Cancer Control (UICC) tumor node metastasis (TNM) staging system is the most reliable indicator of patient prognosis and is widely used among practitioners to determine the most appropriate therapy [3]. However, prognosis may differ substantially even within the same TNM stage. Therefore, new reliable markers are required to improve predictions on the course of disease and lead to a more adjusted treatment. Cancer-related inflammation (CRI) indices, both in the peripheral blood and in the tumor microenvironment, may be suitable for this role. Peripheral systemic inflammatory response (SIR) markers such as LMR, NLR or PLR have potent prognostic value in many malignancies [4,5,6]. The “Immunoscore”—a value based on the density of CD3+ and CD8+ TILs in the tumor center (CT) and the invasive margin (IM)—has been shown to be highly prognostic in colon cancer. Some reports have suggested the superior role of the Immunoscore in predicting survival compared to the TNM staging system [7]. In this study, we tried to evaluate the correlation between local (TILs) and peripheral (LMR, NLR and PLR) CRI biomarkers in CRC patients.

## 2. Materials and Methods

We performed a retrospective analysis of a database of 87 patients treated with radical-intent surgery in the Department of Gastrointestinal Cancers, Maria Skłodowska-Curie National Research Institute of Oncology, Warsaw, Poland, between January 2014 and December 2015. The inclusion criteria were as follows: (1) histologically confirmed cancer of distal sigmoid, rectosigmoid or upper rectum (>10 cm from the anal verge by colonoscopy); (2) no evidence of tumor invading the adjacent organs or distant metastasis; (3) no neoadjuvant treatment applied; and (4) presence of formalin-fixed tissues from the surgical excision of the tumor. The exclusion criteria were: (1) metastatic disease; (2) neoadjuvant chemotherapy and/or radiotherapy; (3) malignant disease of other organs; (4) presence of hematologic malignancies and disorders that could substantially affect inflammatory markers; (5) prior immunosuppressive therapy; and (6) incomplete/inaccurate medical records. Thirty-seven patients were excluded from the study due to the presence of exclusion criteria (10 patients) or the fact that histological specimens were found inadequate for appropriate pathomorphological assessment (27 patients) as shown in Box 1.

Box 1 **Eligibility for the study.**

87 patients with left-sided colorectal cancer (distal sigmoid, rectosigmoid, upper rectum) treated with radical-intent surgery in the Department of Gastrointestinal Cancers, Maria Skłodowska-Curie National Research Institute of Oncology, Warsaw, Poland between January 2014 - December 2015.

Exclusion criteria:

- Metastatic disease found postoperatively-3 patients
- Malignant disease of other organs-1 patient
- Incomplete/Inaccurate medical records-5 patients
- Hematologic malignancies and disorders that could substantially affect inflammatory markers-1 patient.

Histological specimens found inadequate for appropriate pathomorphological assessment–27 patients.

50 patients eligible for the study

We analyzed a routine blood examination before surgery of each patient and calculated their LMR, NLR and PLR by dividing an absolute count of lymphocytes by an absolute count of monocytes, an absolute count of neutrophils by an absolute count of lymphocytes and an absolute count of thrombocytes by an absolute count of lymphocytes in peripheral blood, respectively, as presented in Box 2. The median time between a blood test and the surgery was 3 days (range from 1 to 11 days). The differential white blood cell count was analyzed using the Sysmex XN-550 hematology analyzer following the manufacturer’s protocol.

Box 2 **Calculation of LMR, NLR, PLR.**

Formulas:

LMR—absolute lymphocyte count (109/l) / absolute monocyte count (10 9/l)

NLR—absolute neutrophil count (109/l) / absolute lymphocyte count (10 9/l)

PLR—absolute platelet count (109/l) / absolute lymphocyte count (10 9/l)

The patients were divided in terms of pre-treatment values of SIR markers. The cut-off values were predetermined based on available data in the literature and our previous studies [4,6,8,9]. For LMR, the cut-off value was 2.6, for NLR 3.0 and for PLR 150.

### 2.1. Immunohistochemistry

**Figure 1:**
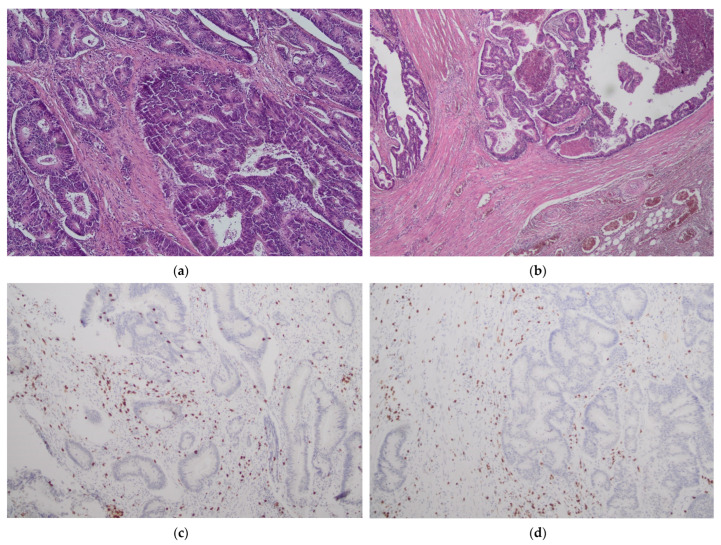
No/minimal lymphocyte infiltrates in tumor center (CT) (**a**,**c**) and tumor margin (IM) (**b**,**d**). H&E staining ×40 (**a**); H&E staining ×100 (**b**); CD3 staining ×100 (**c**,**d**).

**Figure 2:**
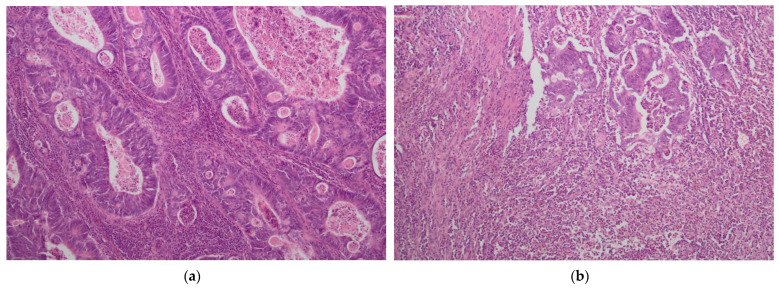

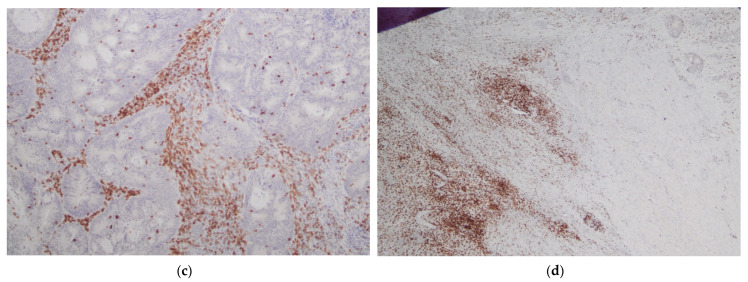
Intense lymphocyte infiltrates in tumor center (CT) (**a**,**c**) and tumor margin (IM) (**b**,**d**). H&E staining ×40 (**a**); H&E staining ×100 (**b**); CD3 staining ×100 (**c**,**d**).

The presence of tumor-infiltrating immune cells in the tumor center and the invasive margin was evaluated by immunohistochemistry using the antibodies for CD3 and CD8 antigens. For immunohistochemical staining, we used primary monoclonal antibodies against CD3 (DAKO, Glostrup, Denmark, Cat. No M7254) and CD8 (DAKO, Denmark, Cat. No IR623) with a DAKO EnVision FLEX detection system (DAKO, Denmark, Cat. No K8002). Paraffin sections (4 µm on silanized slides) were deparaffinized and rehydrated. Antigen epitopes were retrieved by the high temperature method (high pH in PT link). Sections were incubated with primary antibody (20 min), and EnVision FLEX+ target retrieval solution was used. Finally, a color reaction was achieved by incubation with EnVision FLEX DAB chromogen (10 min at room temperature) and hematoxylin counterstain was used for nuclei visualization. Semi-quantitative analysis by an experienced pathologist and a quantitative automated analysis of the specimens was performed. In a semi-quantitative assessment, a four-digit scale (0: 0–10% of the area of scarce and mild staining, 1: 11–50% of the area of moderate or intensive staining, 2: 50–75% of the area of intermediate or intensive staining and 3: >75% area of intermediate or intensive staining) of density of lymphocytes was used in separate measurements for tumor center and invasive margin while in a quantitative assessment an exact number of lymphocytes per 1 mm^2^ of specimen was calculated (using the CellSens Software version 1.16 by Olympus). The highest lymphocyte density regions were selected for histological and immunohistochemical assessment. The representative images of low and high lymphocyte infiltrates in CT and IM are presented in Figure 1 and Figure 2.

### 2.2. Statistical Analysis

The Shapiro–Wilk test was used to test the normality of the data distribution. The correlation between preoperative LMR, NLR, PLR and the density of CD3+ and CD8+ lymphocytes were analyzed using the Spearman’s test. Comparison of parameters between patients according to the stage of the disease was carried out with the Kruskal–Wallis test, while the comparison of TILs according to the pre-treatment value of SIR markers with the Mann–Whitney U test. The Kaplan–Meier survival estimator was calculated, and logrank test was used to compare overall survival for SIR markers and TILs. All statistical analyses were performed using the IBM SPSS Statistics ver. 23 software package. A *p*-value < 0.05 was considered statistically significant.

### 2.3. Ethical Considerations

The study conformed to the provisions of the Declaration of Helsinki and was approved by the ethics committee of Maria Skłodowska-Curie National Research Institute of Oncology in Warsaw.

## 3. Results

Fifty patients were found to be eligible for the study. As presented in Table 1, included in the study were 26 males and 24 females; median age at the initial surgery was 67 years old (range, 44–88 years). The median value of pre-treatment LMR was 3.16 (range 0.95–7.2). The medians of NLR and PLR were 2.34 (0.7–14.54) and 140 (58–358), respectively.

Resected specimens were pathologically classified according to the UICC TNM classification of malignant tumors, ver. 7. The distribution of cancer stages was as follows: stage I-11/50 (22%); stage II–18/50 (36%); stage III-21/50 (44%) patients. Tumor grade was mostly G2 (32 cases), followed by G1 (10) and G3 (5). In three cases, the grade remained indeterminate.

The densities of CD3+ and CD8+ lymphocytes were evaluated in the CT and IM of the resected tumors. In three cases, CD3+ and CD8+ lymphocytes were detected only in the invasive margin and not in the center of the specimen. The mean density of CD3+ lymphocytes per 1 mm^2^ was 1699 (range 704–3900) in CT and 1929 (368–4959) in IM. The densities of CD8+ lymphocytes in CT and IM were 877 (66–3918) and 1255 (175–2511), respectively.

There was a statistically significant correlation between the density of both CD3+ and CD8+ cells in CT and IM (*p* ≤ 0.005), Table 2.

Median value of SIR markers and TILs have been evaluated according to the stage of the disease. No statistically significant differences in the level of parameters between the stages of the disease were found, as presented in Table 3 (*p* > 0.05 in all cases).

We found no correlation between pre-treatment LMR, NLR, PLR and the density of CD3+ and CD8+ TILs, Table 4.

### The Semi-Quantitative Evaluation

There were no significant differences in the average level of semi-quantitative evaluation of CD3+ and CD8+ lymphocytes between groups of pre-treatment LMR above/below 2.6, NLR above/below 3.0 and PLR above/below 150, as presented in Table 5.

## 4. The Quantitative Evaluation

In the quantitative assessment, level of CD3+ in CT was significantly higher in NLR < 3.0 than NLR ≥ 3.0 patients, median of 1767.86 (range: 705.36–3900.00) vs. 1233.93 (703.57–2950.00), respectively (*p* = 0.044). No differences in the average level of quantitative evaluation of CD3+ and CD8+ lymphocytes between the groups of pre-treatment LMR above/below 2.6 and PLR above/below 150 were found, as presented in Table 5.

### The Analysis of Overall-Survival (OS)

Patients participating and eligible for the study treated in Maria Sklodowska-Curie National Research Institute of Oncology between March 2014 and September 2015 were followed-up until December 21, 2020. The median follow-up time was over 6 years.

**Figure 3:**
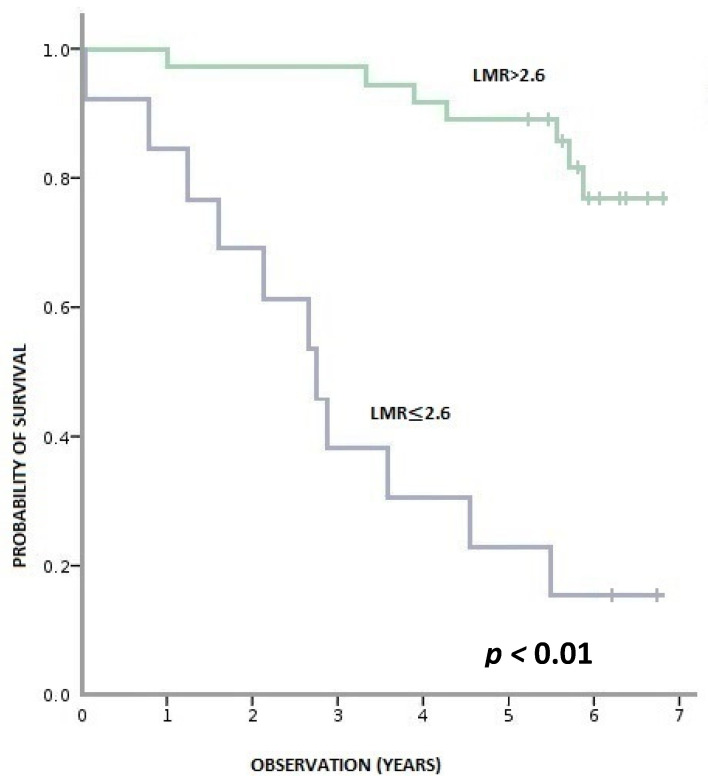
Overall survival according to pre-treatment LMR (lymphocyte-to-monocyte ratio).

**Figure 4:**
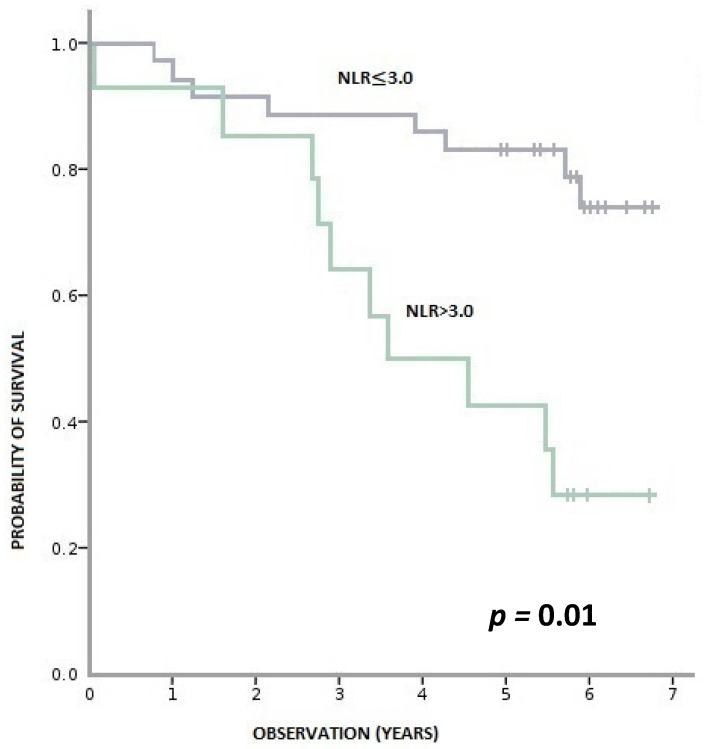
Overall survival according to pre-treatment NLR (neutrophil-to-monocyte ratio).

**Figure 5:**
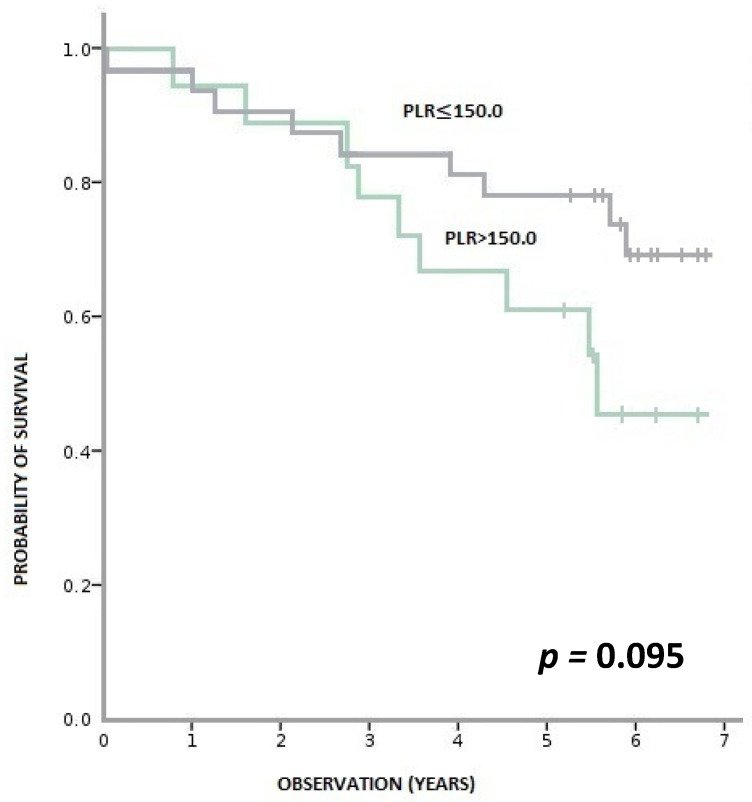
Overall survival according to pre-treatment PLR (platelet-to-lymphocyte ratio).

During this time, 36% (18/50) of patients died, and 64% (32/50) remained alive. Patients with pre-treatment LMR > 2.6 had a statistically significant longer OS than patients with LMR ≤ 2.6 (*p* < 0.001). Similarly, patients with baseline NLR > 3 had a statistically shorter OS than those with NLR ≥ 3 (*p* = 0.001). We observed a tendency towards better OS in patients with PLR ≤ 150 compared to PLR > 150; however, the result was statistically insignificant (*p* = 0.095) (Figure 3, Figure 4 and Figure 5).

**Figure A1:**
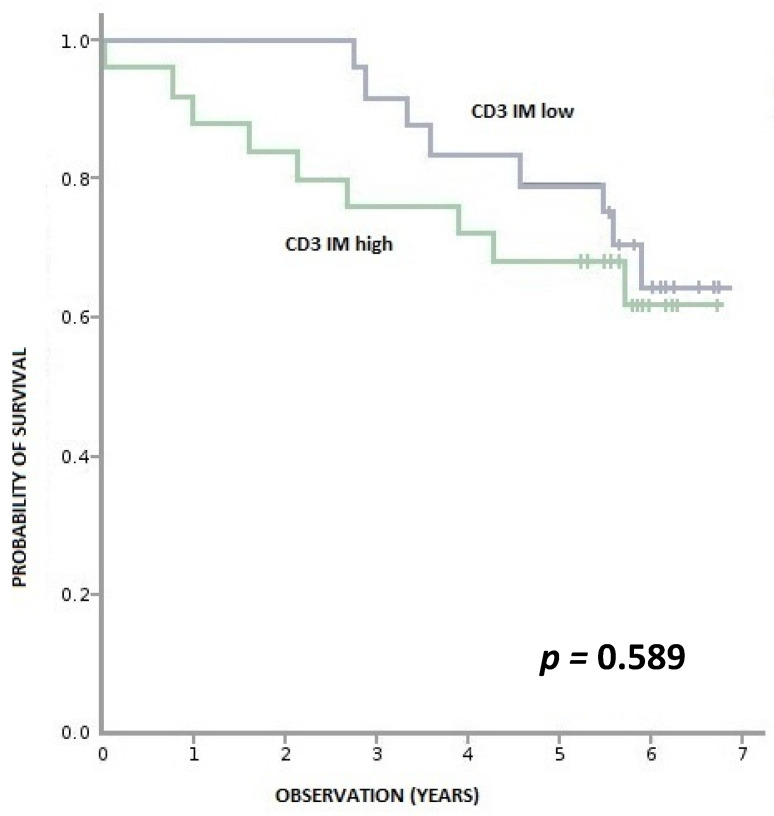
Overall survival according to density of CD3+ lymphocytes in IM (invasive margin). CD3+ IM high—≥ 970 per 0.56 mm^2,^ CD3+ IM low—< 970 per 0.56 mm^2^.

**Figure A2:**
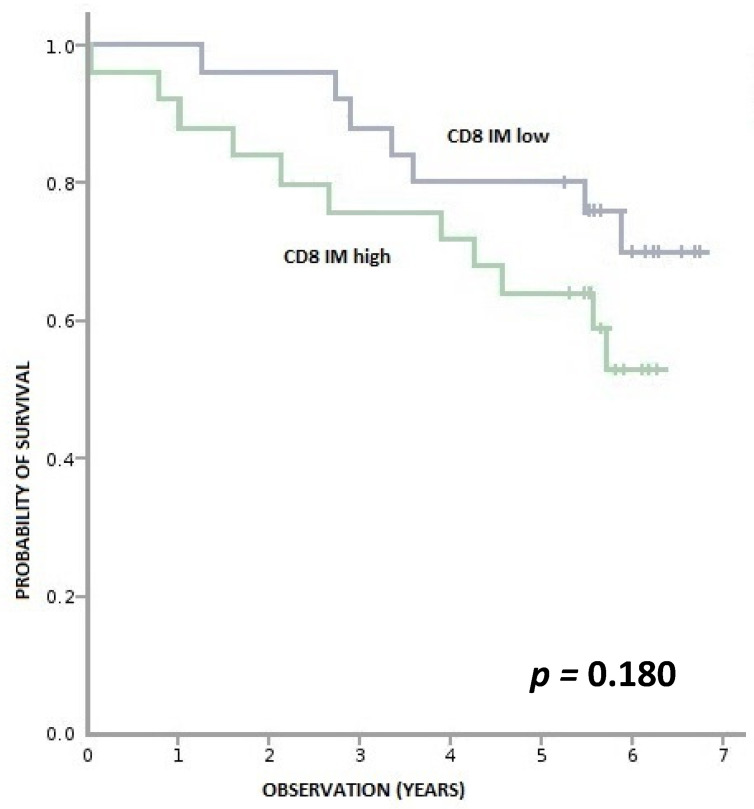
Overall survival according to density of CD8+ lymphocytes in IM (invasive margin). CD8+ IM high—≥ 670 per 0.56 mm^2,^ CD8+ IM low—<670 per 0.56 mm^2^.

**Figure A3:**
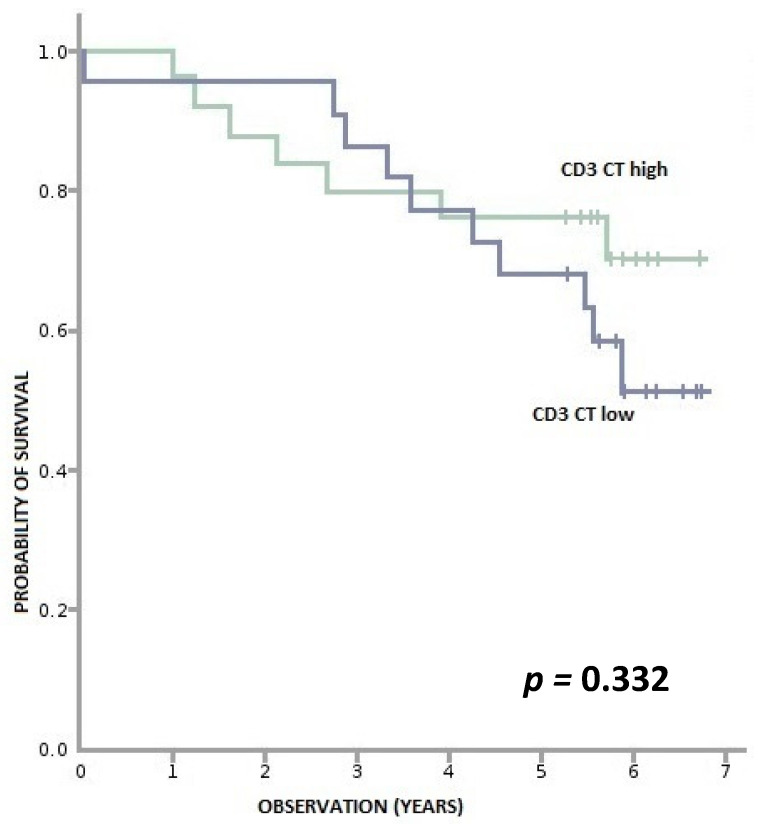
Overall survival according to density of CD3+ lymphocytes in CT (tumor center). CD3+ CT high—≥ 840 per 0.56 mm^2,^ CD3+ CT low—<840 per 0.56 mm^2^.

**Figure A4:**
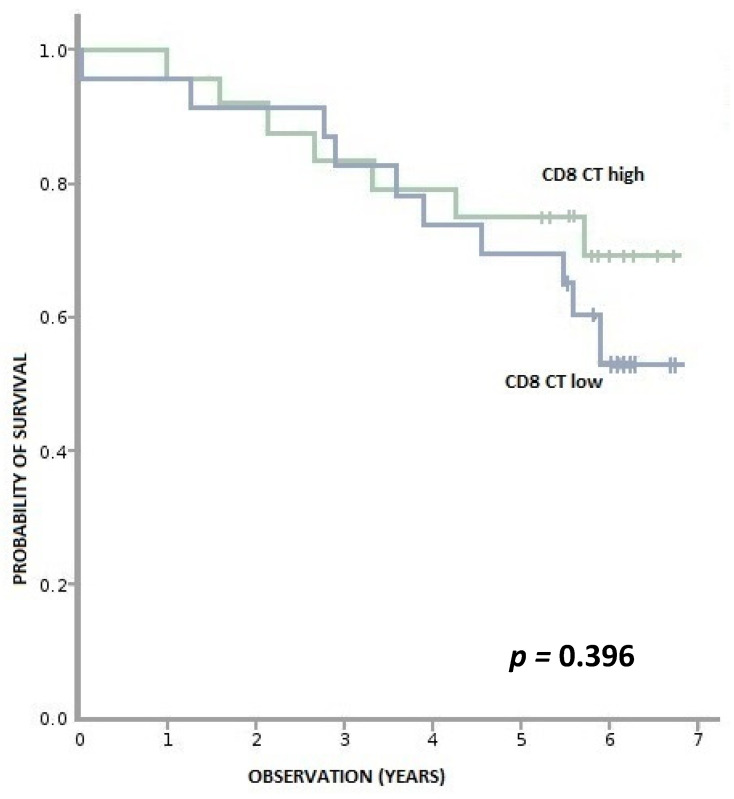
Overall survival according to density of CD8+ lymphocytes in CT (tumor center). CD8+ CT high—≥ 404 per 0.56 mm^2,^ CD8+ CT low—< 404 per 0.56 mm^2^.

We found no correlation between levels of CD3+ and CD8+ lymphocytes in the cancer tissue and OS (Appendix A, Figure A1, Figure A2, Figure A3 and Figure A4).

## 5. Discussion

Our hypothesis was that there is a correlation between peripheral and local CRI markers and that SIR markers such as LMR, NLR and PLR may act as peripheral blood-based surrogates of TILs. The goal was to analyze the relationship between these parameters in left-sided CRC. Few studies have investigated this subject, and available data is scarce. The definition of TILs varies between studies as often different types of lymphocytes (CD3+, CD4+, CD5+, CD8+, CD45RO+, etc.) are taken into account and scored accordingly to different gradings. Moreover, there are no established cut-off values for SIR markers or TILs.

We observed a higher number of immune cells in IM than in CT and a positive correlation between the densities of CD3+ and CD8+ cells in both tumor regions, which is in accordance with available data from other reports.

Mean values of CD3+ and CD8+ lymphocytes in our study were high compared to some analyses; however, even higher mean densities of lymphocytes, especially in early stages of the disease, and higher intrapatient variability of the density of lymphocytes have been reported [10,11,12,13]. In most studies, only semi-quantitative evaluations of TIL densities were conducted, making it difficult to perform conclusive comparisons in this regard. Our analysis showed that patients with a pre-treatment value of NLR < 3.0 had a statistically significantly higher level of CD3+ lymphocytes in the center of the resected tumor compared to the NLR-high group. However, based on Spearman’s rho test, no correlation between pre-treatment values of LMR, NLR and PLR and the density of CD3+ or CD8+ TILs was observed. The results of other studies concerning the relation between LMR, NLR, PLR and density of TILs are conflicting. Kwan Ho Lee et al. assessed a relationship between TILs and hematologic parameters in breast cancer. A statistically significant correlation between lymphocyte and monocyte count, LMR and CD8+ TILs has been demonstrated [14]. In another study, high TILs (CD3+, CD15+ and CD68+) were significantly correlated with low NLR and high LMR in locally advanced triple-negative breast cancer [15]. High preoperative NLR was associated with low TILs in hepatocellular carcinoma [16]. In patients who underwent curative surgery for gastric cancer, CD3+ and CD8+ immune cells densities were not associated with pre-treatment NLR [17]. A negative correlation between NLR and CD3+ was detected in patients with non-small cell lung cancer [18]. In CRC, a relationship between TILs and SIR markers has been observed mainly indirectly throughout common prognostic properties [19,20]. The results of more direct correlations are scarce and unclear. In a study by Guo et al., high values of LMR were associated with a high intratumoral number of CD3+ T cells in CT. However, no correlations between either LMR and CD3+ T-cells in IM or between CD3+ T-cells and NLR or PLR were found. No correlations between CD8+ lymphocytes and LMR, NLR or PLR were detected either [21]. In a study evaluating rectal cancer patients, there was no correlation between baseline NLR and CD8+ lymphocytes; CD3+ T-cells, LMR or PLR were not evaluated [22].

Our study focused on investigating the association between inflammatory markers in circulating blood and tumor tissue rather than on prognostic outcomes; we did, however, perform an analysis of the association between peripheral and local CRI markers and OS. Despite the fact that there was no correlation between peripheral and local CRI markers and disease stage, strong prognostic values of LMR, NLR and, to a lesser extent, PLR were confirmed among our patients in accordance with most studies [23,24,25]. A correlation between the density of TILs and OS was, nonetheless, not observed. This phenomenon is not consistent with the majority of other results [26,27]. However, a number of studies also failed to show the expected correlation—entirely or, at least, in some of the analyzed cohorts [28,29,30,31]. It is speculated that the lack of correlation between density of TILs and survival or other prognostic factors (e.g., stage of disease) may be due to environmental variables, such as the microbiome or tumor inflammatory status [32,33].

Possibly, the differences in tumor biology and immune microenvironment may affect the severity of the impairment of T-cells and the sensitivity of tumor cells to their cytotoxic functions affecting the immunological response [30].

In order to thoroughly understand the role and potential of peripheral and local inflammatory indices, it is crucial to comprehend the impact of each of their components on CRI. The relationship between inflammation and cancer is well-proven. This phenomenon was first discovered in the 19th century by observing inflammatory cells in resected tumor tissues and associated sites of chronic inflammation with carcinogenesis [34]. CRI affects many aspects of malignancy. It involves not only a reaction of the host’s immune system against the tumor, but also inflammatory chemokines and cytokines released by tumor-associated leukocytes and cancer cells contributing to tumor growth, invasion and metastatic activity [35]. Lymphocyte count reflects the responsiveness of the immune system of the host. Lymphocytes inhibit cancer proliferation and spread [36]. Lymphopenia is often observed in advanced cancer and may result in a weak and insufficient immunological response. Studies have linked it with unfavorable prognosis in oncological patients [37]. By contrast, monocytes, neutrocytes and platelets play a vital role in tumor progression [38,39,40]. A correlation between monocytosis and a poor prognosis has been reported in many cancers [41]. Neutrophils, as the most common subset of leukocytes, have a substantial impact on CRI. They have been shown to play an important role in the initiation and progression of cancer [42]. Like monocytes, a high count of neutrophils has been associated with unfavorable outcomes in many malignancies [43]. Similarly, an elevated level of thrombocytes has been linked to a poor prognosis. Tumor cells, through cytokines and interleukins, stimulate megakaryocytes to induce the production and activation of thrombocytes. In turn, thrombocytes release angiogenic and growth factors, such as vascular endothelial growth factor and platelet-derived growth factor, substantially contributing to angiogenesis and tumor growth [44,45]. Peripheral blood-based biomarkers, such as LMR, NLR and PLR, take advantage of the combined prognostic value of the mentioned blood components. CRI affects tumor microenvironments to a large extent. Tumor-infiltrating immune cells (TIICs) are composed mainly of immunological cells, such as tumor-associated macrophages, dendritic cells, mast cells and lymphocytes. TILs—white-blood cells originating from the bloodstream, which migrated towards the tumor site—are the most investigated subpopulation of TIICs. TILs are involved in the recognition and elimination of tumor cells and play an important role in boosting anti-tumor immunity [46]. Their prognostic and predictive role is well-established in breast cancer, especially the TNBC subtype, where a high level of TILs is correlated with better OS, disease-free survival and higher pathological complete response rate following neoadjuvant therapy [47,48]. TILs are also correlated with an improved prognosis in several other cancers, such as lung, ovarian and pancreatic [49,50,51]. In CRC, an Immunoscore—a classification evaluating two lymphocyte populations (CD3+/CD45RO+, CD3+/CD8+ or CD8+/CD45RO+)—both in the CT and IM was developed. According to some reports, the Immunoscore is a superior predictor of OS and DFS compared to the AJCC/UICC TNM classification system in CRC [7].

In designing our study, we based it on the Immunoscore—we evaluated the quantitative and semi-quantitative density of populations of CD3+ and CD8+ lymphocytes in CT and IM. We focused on cancers of left-sided colon (distal sigmoid and rectosigmoid) and the upper part of the rectum, excluding middle and low rectal cancers, as it was essential for us to avoid any presurgical treatment (radio or chemotherapy) that could influence inflammation indices both in peripheral blood and in the tumor tissue. Low and middle rectal cancers, unlike those in the upper rectum and colon, are often treated with neoadjuvant radio/radio-chemotherapy. It is important to note that we decided to study tissues from the surgical excision of the tumor, not the presurgical biopsies, as standard biopsies often do not include an invasive margin area, which is the primary site of interaction between malignant and immune cells [10]. Surgical specimens, however, allow an ample evaluation of tissue sections, especially with regard to invasive margins. The main limitation of our study was the small number of patients. The study was also retrospective, and we cannot exclude some patients that had non-cancer-related inflammation that could have affected the outcomes. Therefore, the results need to be repeated in bigger cohorts in prospective trials. However, it is worth noting that we conducted a novel study on the subject that is surprisingly absent in the literature. We hope our study will attract more attention to the topic and encourage further investigation.

## 6. Conclusions

Both blood-based SIR markers and tumor-infiltrating immune cells have been thoroughly investigated in recent years as prognostic factors in many cancers. They reflect a peripheral and local aspect of the immunological reaction within the CRI. To our knowledge, our study is the first to evaluate a direct correlation between LMR, NLR, PLR and TILs in left-sided colorectal cancer. We found that a level of CD3+ lymphocytes in the center of the tumor was significantly higher in patients with low pre-treatment NLR; however, no correlation between any of the pre-treatment blood-based markers and CD3+ or CD8+ lymphocytes in the resected tumor was demonstrated. Further investigations on a larger scale are crucial in order to better understand this relation.

## Figures and Tables

**Table 1:** Characteristics.

Characteristic	All Patients (*n* = 50)
Age (years), median (range)	67 (44–88)
Sex, n (%)	
Male	26 (52.0)
Female	24 (48.0)
Tumor, n (%)	
T1-T2	13 (26.0)
T3-T4	37 (74.0)
Lymph nodes, n (%)	
N0	29 (58.0)
N1-N2	21 (42.0)
Grade, n (%)	
G1	10 (20.0)
G2	32 (64.0)
G3	5 (10.0)
Gx	3 (6.0)
Stage, n (%)	
I	11 (22.0)
II	18 (36.0)
III	21 (42.0)
ALC (10^9/l), median (range)	1.94 (0.69–3.95)
AMC (10^9/l), median (range)	0.57 (0.30–1.14)
ANC (10^9/l), median (range)	4.10 (2.01–10.03)
Platelets (10^9/l), median (range)	246 (153–430)
LMR, median (range)	3.16 (0.95–7.20)
NLR, median (range)	2.34 (0.70–14.54)
PLR, median (range)	140 (58–358)
CD3 CT/mm^2^, mean (range)	1699 (704–3900)
CD3 IM/mm^2^, mean (range)	1929 (368–4959)
CD8 CT/mm^2^, mean (range)	877 (66–3918)
CD8 IM/mm^2^, mean (range)	1255 (175–2511)

ALC, absolute lymphocyte count; AMC, absolute monocyte count; ANC, absolute neutrophil count; LMR, lymphocyte-to-monocyte ratio; NLR, neutrophil-to-monocyte ratio; PLR, platelet-to-lymphocyte ratio; CT, tumor center; IM, invasive margin.

**Table 2:** Correlation between CD3+ and CD8+ TILs in CT and IM.

TILs	CD3 CT		CD3 IM		CD8 CT		CD8 IM	
	r	*p*	r	*p*	r	*p*	r	*p*
CD3 CT	x	x	0.52	<0.001	0.52	<0.001	0.40	0.005
CD3 IM	0.52	<0.001	x	x	0.59	<0.001	0.69	<0.001
CD8 CT	0.52	<0.001	0.59	<0.001	x	x	0.59	<0.001
CD8 IM	0.40	0.005	0.69	<0.001	0.59	<0.001	x	x

TILs, tumor-infiltrating lymphocytes; CT, tumor center; IM, invasive margin; r, correlation coefficient calculated with Spearman’s rho test.

**Table 3:** Comparison of SIR markers and TILs according to the stage of the disease.

SIR Markers/TILs	Stage I	Stage II	Stage III	*p*
LMR	2.88 (1.13–7.20)	3.42 (2.30–6.00)	3.00 (0.95–6.23)	0.501
NLR	3.66 (0.70–13.09)	2.15 (1.20–4.64)	2.17 (0.97–14.54)	0.579
PLR	135.00 (58.00–268.00)	140.00 (61.00–187.00)	140.00 (66.00–358.00)	0.910
CD3 CT/mm^2^	1602 (704–2745)	1771 (705–3336)	1687 (716–3900)	0.829
CD3 IM/mm^2^	1795 (636–2818)	1913 (368–3920)	2006 (900–4959)	0.965
CD8 CT/mm^2^	939 (66–3918)	973 (432–3445)	734 (161–2664)	0.226
CD8 IM/mm^2^	1042 (236–1827)	1302 (232–2511)	1325 (175–2432)	0.464

SIR, systemic inflammatory response; TILs, tumor-infiltrating lymphocytes; LMR, lymphocyte-to-monocyte ratio; NLR, neutrophil-to-monocyte ratio; PLR, platelet-to-lymphocyte ratio; CT, tumor center; IM, invasive margin. Data presented as median (range). Groups compared with Kruskal–Wallis test.

**Table 4:** Correlation between LMR, NLR, PLR and CD3+ and CD8+ TILs in CT and IM.

TSIR Markers	CD3 CT		CD3 IM		CD8 CT		CD8 IM	
	r	*p*	r	*p*	r	*p*	r	*p*
LMR	0.08	0.575	0.03	0.857	0.19	0.195	0.03	0.854
NLR	0.05	0.720	0.08	0.606	0.10	0.496	0.03	0.843
PLR	0.07	0.645	0.12	0.424	0.03	0.831	0.09	0.518

SIR, systemic inflammatory response; LMR, lymphocyte-to-monocyte ratio; NLR, neutrophil-to-monocyte ratio; PLR, platelet-to-lymphocyte ratio; CT, tumor center; IM, invasive margin; r, correlation coefficient calculated with Spearman’s rho test.

**Table 5:** Comparison of TILs according to pre-treatment values of SIR markers.

TILs	LMR			NLR			PLR		
	LMR ≤ 2.6	LMR > 2.6	p	NLR ≥ 3.0	NLR < 3.0	p	PLR ≥ 150	PLR < 150	p
s-q CD3 CT	1 (0–3)	2 (0–3)	0.287	2 (0–2)	1 (0–3)	0.909	2 (0–3)	1 (0–3)	0.553
q CD3 CT/mm^2^	1201.79 (703.57–3 900.00)	1544.64 (705.36–3 335.71)	0.213	1233.93 (703.57–2 950.00)	1767.86 (705.36–3 900.00)	0.044	1289.29 (703.57–3 107.14)	1517.86 (705.36–3 900.00)	0.868
s-q CD3 IM	1 (0–3)	2 (0–3)	0.076	1 (0–3)	2 (0–3)	0.204	1 (0–3)	2 (0–3)	0.760
q CD3 IM/mm^2^	1560.71 (1 112.50–4 958.93)	1783.93 (367.86–3 919.64)	0.688	1541.07 (367.86–2 248.21)	1971.43 (635.71–4 958.93)	0.061	1682.14 (367.86–3 919.64)	1783.93 (635.71–4 958.93)	0.862
s-q CD8 CT	0 (0–2)	1 (0–3)	0.199	1 (0–2)	1 (0–3)	0.807	1 (0–3)	1 (0–2)	0.579
q CD8 CT/mm^2^	566.07 (160.71–2 664.29)	787.50 (66.07–3 917.86)	0.196	727.68 (160.71–1 266.07)	721.43 (66.07–3 917.86)	0.879	789.29 (171.43–3 917.86)	682.14 (66.07–3 444.64)	0.682
s-q CD8 IM	1 (0–3)	1 (0–3)	0.317	1 (0–2)	1 (0–3)	0.448	1 (0–3)	1 (0–2)	0.533
q CD8 IM/mm^2^	1196.43 (553.57–2 432.14)	1119.64 (175.00–2 510.71)	0.930	1075.89 (564.29–1 826.79)	1208.93 (175.00–2 510.71)	0.790	1196.43 (523.21–2 510.71)	1158.04 (175.00–2 330.36)	0.440

TILs, tumor-infiltrating lymphocytes; s-q, semi-quantitative; q, quantitative; LMR, lymphocyte-to-monocyte ratio; NLR, neutrophil-to-monocyte ratio; PLR, platelet-to-lymphocyte ratio; CT, tumor center; IM, invasive margin. Data presented as median (range). Groups comparison with Mann–Whitney U test.

## Data Availability

The data presented in this study are available on request from the corresponding author. The data are not publicly available due to medical data privacy issues.
